# Recent progress of Rh‐based three‐way catalysts

**DOI:** 10.1002/smo.20240004

**Published:** 2024-06-11

**Authors:** Ruize Jiang, Huilin Wang, Li Liu, Baokang Geng, Xiang Chu, Yi Shi, Xiao Wang, Shuyan Song, Hongjie Zhang

**Affiliations:** ^1^ State Key Laboratory of Rare Earth Resource Utilization Changchun Institute of Applied Chemistry Chinese Academy of Sciences Changchun China; ^2^ School of Applied Chemistry and Engineering University of Science and Technology of China Hefei China; ^3^ Department of Chemistry Tsinghua University Beijing China

**Keywords:** mechanism and kinetics, rhodium (Rh) catalysts, structure‐activity relationships, three‐way catalysts

## Abstract

Three‐way catalysts are widely used to control criterion pollutant emissions from the increasing gasoline engines. With the stringent requirements of automotive pollutant emission standards in various countries, Rh has become an irreplaceable component of three‐way catalysts due to its superior NO_x_ elimination, high N_2_ selectivity, and simultaneous elimination of CO and hydrocarbons. In this review, we systematically review the recent development of Rh‐based three‐way catalysts in terms of potential supports and effective active center construction strategies. We further summarize the key role of Rh metal in the three‐way catalytic mechanism and reaction kinetics. Finally, we conclude the current challenges and future opportunities facing Rh‐based catalysts. It is believed that based on the deep understanding of Rh‐based three‐way catalysts, the design of Rh‐based catalysts with good low‐temperature catalytic performance and low cost is expected to be realized in the future.

## Graphical abstract

In this paper, we generalized the progress of Rh‐based three‐way catalysts in recent decades from the aspect of the support property and the active sites construction. Subsequently, we elucidate the three‐way reaction mechanism as well as the intrinsic reason leading to deactivation. Finally, we present the exciting challenges and future development of Rh‐based three‐way catalysts.

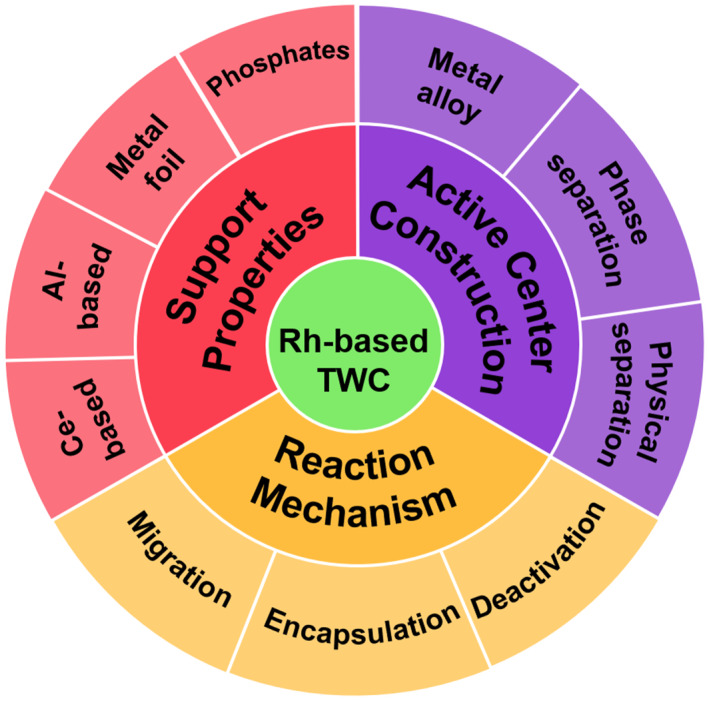

## 1 INTRODUCTION

With the spread of industrialization, automobiles have become one of the most widely used means of transportation.[^1^, ^2^, ^3^] Vehicle exhaust is a major source of air pollution, containing pollutants such as hydrocarbons (HC), carbon monoxide (CO) and nitrogen oxides (NO_x_), which result in global environmental problems such as global warming, ozone layer depletion and acid rain. Three‐way catalysts are widely used in petrol‐powered vehicle exhaust emissions, because they can effectively convert the CO, HC, and NO_x_ pollutants into harmless products (CO_2_, N_2_, and H_2_O).^[4]^ Many countries have introduced corresponding policies to limit automobile exhaust emissions, such as the European Commission's Real World Driving Emissions testing program, China's VI emission standards, and India's Bharat Stage VI standards, which require the use of three‐way catalysts in all vehicles.[^5^, ^6^] With the seriousness of the pollution problem, the standards of governance policies are gradually rising. Newly introduced emissions regulations are also being progressively tightened to require a higher percentage of new vehicles to meet more stringent ultra‐low emission vehicle standards.[^7^, ^8^] These policies have not only reduced the use of fossil energy and improved people's health in terms of controlling air pollution but also promoted the innovation and upgrading of automobile exhaust treatment technologies. Currently 98% of vehicles are equipped with three‐way catalysts, and with the increase in vehicle emission standards, it is foreseeable that this percentage will reach 100% in the future.^[9]^ So, the development of high‐performance and low‐cost three‐way catalysts has important application prospects.

In general, a three‐way catalyst consists of a support, an active alumina coating, some additives, and active components.^[10]^ The common supports for three‐way catalysts are often cordierite and metal, which provide a high specific surface area favorable for loading active sites.^[11]^ The additives are mainly cerium‐based oxides, which can effectively widen the air‐fuel ratio, enhance the antitonicity, inhibit high‐temperature sintering, and improve pollutant conversion.^[12]^ The active components are the core of the three‐way catalysts and play an important role in the catalytic process. The commonly used active components are primarily platinum group metals (Pt, Rh, and Pd).[^13^, ^14^] Among them, Pt and Pd have strong adsorption ability toward CO and O_2_ and thus are mostly involved in the oxidation reaction in the three‐way catalysts, as shown in Equations ((1), (2), (3), (4)).

(1) $$C_{m}H_{n}+O_{2}\;\to \;{CO}_{2}\;\left(CO\right)+H_{2}O$$

(2) $$CO+O_{2}\;\to \;{CO}_{2}$$

(3) $$CO+H_{2}O\;\to \;{CO}_{2}+H_{2}$$

(4) $$H_{2}+O_{2}\;\to \;H_{2}O$$

In 1978, Hegedus first discovered the critical role of Rh as an active component in three‐way catalysts for NO_x_ elimination. Subsequently in the 1980s, the U.S. Environmental Protection Agency imposed stringent controls on NO_x_ emissions, which led to the phase‐out of previous platinum‐ and palladium‐based catalysts as they were inadequate to meet market demand.[^15^, ^16^] Compared to Pt and Pd, Rh has a superior NO adsorption and dissociation capacity, which is considered as the first step of NO degradation. A suitable Rh center also increases N_2_ selectivity and reduces harmful byproducts such as NH_3_ and N_2_O.[^17^, ^18^] Besides, the addition of Rh can also improve the low‐temperature activity of the three‐way catalysts because NO_x_ can be used as oxidants to further facilitate the elimination of CO and hydrocarbons.[^17^, ^18^, ^19^] The corresponding reactions are shown in Equations ((5), (6), (7)).

(5) $$C_{m}H_{n}+NO\;\to \;N_{2}\;\left(N_{2}O,{NH}_{3}\right)+H_{2}O+{CO}_{2}$$

(6) $$CO+NO\;\to \;{CO}_{2}+N_{2}\;\left(N_{2}O\right)$$

(7) $$H_{2}+NO\;\to \;N_{2}\;\left(N_{2}O,{NH}_{3}\right)+H_{2}O$$

**FIGURE 1:**
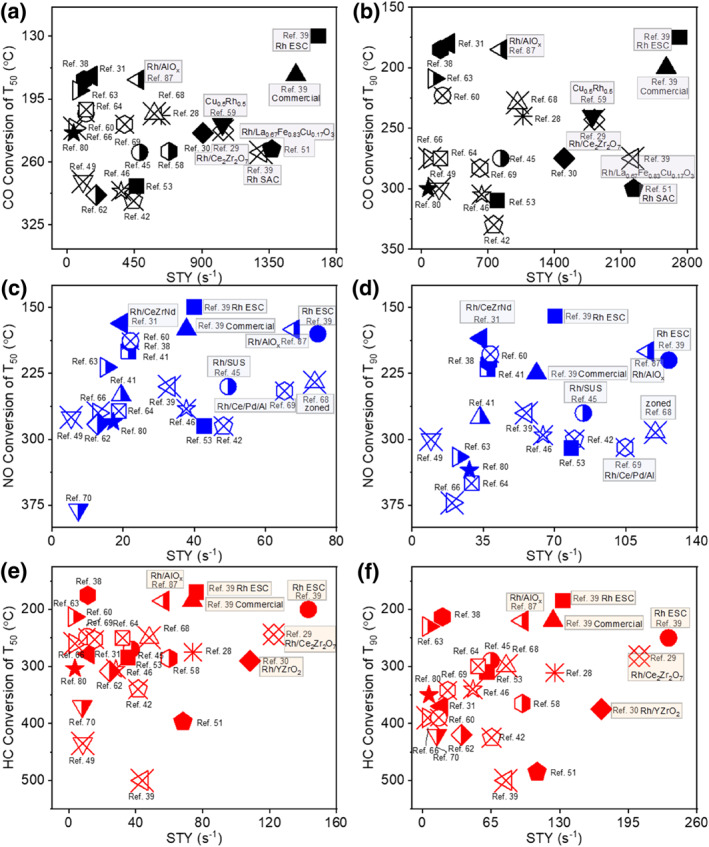
Comparison of T_50_ values (a, c, and e) for CO, HC, and NO and T_90_ values (b, d, and f) for CO, HC, and NO based on the reported performance of Rh‐based catalysts.

Due to the scarcity of Pt group metals, improving the utilization efficiency of noble metals has been an important topic in three‐way catalyst research. Compared with Pt and Pd, Rh is less abundant and more expensive, thus reducing the content of Rh in three‐way catalysts is considered to have more potential to reduce the cost of the whole three‐way catalyst.[^20^, ^21^] Currently, the major approaches to promote the utilization efficiency of Rh include: (i) dispersing Rh onto supports with larger specific surface area to improve the mass transfer efficiency; (ii) preparing Rh single‐atom catalysts to improve the atomic utilization; modifying metal‐support interactions to change the electronic structure of noble metals and to enhance the low‐temperature activity; (iii) coupling another metal with lower cost to enhance the catalytic activity and reduce the Rh content.^[22]^ To provide guidance for future high‐performance three‐way catalysts, this review summarizes the advanced progress of Rh‐based three‐way catalysts in recent years. Rh‐based three‐way catalysts contain not only single Rh sites but also multi‐metal sites, and it is difficult to compare multi‐metal site catalysts by traditional turnover frequency (TOF). Therefore, we calculated the rate for converting pollutants from noble metals by space‐time yield (STY), as shown in Equation (8). The acronym STY represents the gas conversion capacity per unit time of a noble metal mass. The single Rh atom catalysts which used cerium‐based, aluminum‐based, and perovskite supports exhibited significantly high STY. Alloy‐based catalysts typically exhibit a slightly lower STY due to the inefficient utilization of Rh, but this can be improved by reducing the Rh content or tuning the electronic properties. A higher STY signifies an enhanced capacity to eliminate pollution per unit mass of noble metal, and conversely (Figure 1). We found that the activity of Rh‐based catalysts depends on the fine structure of the whole catalyst. Minor alterations in the support or active center can result in a significant increase in three‐way catalytic performance. Therefore, we summarized the role of Rh components on different supports and the synergy between Rh and other metals. Based on the deep understanding of the structure of Rh‐based catalysts, we further studied the mechanism as well as the kinetic behavior of different Rh sites in the three‐way catalysis, especially in the CO + NO reaction. Finally, we concluded the challenges facing Rh‐based three‐way catalysts and provided an outlook for future Rh‐based catalysts with low cost and high catalytic activity. We hope that this review will ultimately inspire the structure‐based design of advanced Rh‐based catalysts.

(8) $$STY=\frac{GHSV\times p^{ө}\times \omega_{gas}\times n_{conv.}}{R\times T\times \omega_{metal}}\;\left(s^{-1}\right)$$

GHSV is gas hourly space velocity, *p^ɵ^* is standard atmospheric pressure, R is gas constant, *ω*_gas_ and *ω*_metal_ represent the concentration of gas (CO, NO, or HC) and the total mass content of noble metals (Pt, Rh, and Pd), respectively, *n*_conv._ represents the substrate conversion rate, and T represents the temperature, T_50_ and T_90_ represent the temperatures at which the substrate conversion rate reaches 50% and 90%, respectively. All units are in the international system of units.

## 2 ROLE OF SUPPORT PROPERTIES IN RH‐BASED CATALYSTS

The support is an intrinsic part of the three‐way catalysts, and plays an important role in regulating catalytic activity. Properly regulated supports in catalytic reactions will have a facilitating effect on catalytic reactions, for example, a large surface area is beneficial to improve the mass transfer efficiency, proper metal‐support interactions are helpful to enhance the stability of the metal‐active sites, electron transfer between the supports and active sites is favorable for the adsorption enhancement of gas molecules, and the unique oxygen storage capacity of the supports is friendly to the conduct of redox reactions.[^23^, ^24^] In this section, we summarize several types of cerium‐based supports, aluminum‐based supports, metal foil supports, and other supports. Then, we discuss the advantages of each support in anticipation of finding the commonality of supports suitable for three‐way catalysts.

### 2.1 Cerium‐based supports

Since the 1980s, cerium oxide has been introduced into commercial catalysts due to its redox properties, stable loading of noble metals, metal‐support interactions, and oxygen storage capacity.^[25]^ Previous studies pointed out that, cerium‐based support can effectively expand the air‐fuel ratio, improve the toxicity resistance, and inhibit the sintering of active centers during high‐temperature operation. Moreover, Rh‐loaded Ce‐based catalysts often exhibit good low‐temperature pollutant conversion due to their suitable pore structure, appropriate surface acidity and alkalinity, and optimized chemical valence.^[26]^

**FIGURE 2:**
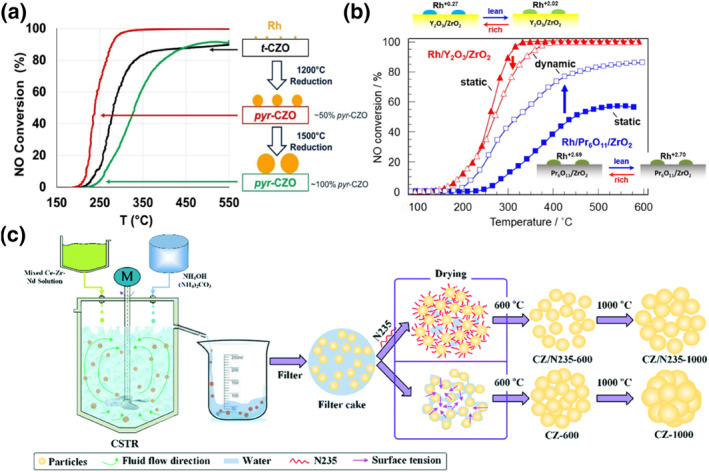
(a) Schematic of Rh‐loaded pyrochlore phase cerium‐zirconium.^[30]^ Reproduced with permission.^[30]^ Copyright 2021, Elsevier. (b) Schematic of Y and Pr doped ZrO_2_.^[31]^ Reproduced with permission.^[31]^ Copyright 2017, Elsevier. (c) Schematic of Nd doped cerium‐zirconium solid solution.^[32]^ Reproduced with permission.^[32]^ Copyright 2016, Royal Society of Chemistry.

Exposure of catalysts to elevated temperatures can easily lead to sintering of the Ce‐based support and active noble metals, which may result in loss of oxygen storage capacity (OSC) and degradation of catalytic performance.^[27]^ Among these compounds, cerium‐zirconium solid solution materials have attracted much attention due to their good weaving properties, excellent thermal stability, and redox properties. To improve the stability of Rh‐loaded cerium‐zirconium solid solutions, tuning the physical phase of the support is an interesting avenue.[^28^, ^29^] Wu and his co‐workers found that the pyrochlore‐phase Ce_2_Zr_2_O_7_ obtained by reduction of the corresponding cubic‐phase solid solution at 1200°C retained a good OCS‐promoting effect (Figure 2a).^[30]^ The pyrochlore phase was similar to the pre‐sintered support, which avoided the sintering deactivation of the subsequently loaded Rh species. While Rh in the cubic‐phase ceria‐zirconia solid solution has the strongest metal‐support interactions leading to catalyst deactivation. Loading the chlorite phase with a highly dispersed 0.1% Rh effectively avoids deactivation due to strong metal‐support interactions. Compared to the cubic phase samples, pyrochlore phase samples showed a decrease in T_50_ values for NO and HC by 44°C and 48°C, respectively. In addition to the modification of the support phase and morphology, another effective common strategy is the introduction of trivalent rare earth ions (Cerium (Ce^4+^), Lanthanum (La^3+^), Neodymium (Nd^3+^), Praseodymium (Pr^3+^) and Yttrium (Y^3+^)) into supports. Haneda and his co‐workers modified the surface of ZrO_2_ using Ce‐based additives and found that the rare earth additives can control the surface alkalinity and stabilize Rh species in a highly dispersed state (Figure 2b).^[31]^ They pointed out that the Y_2_O_3_ is the best additive for Rh/ZrO_2_, and Rh species on Rh/Y_2_O_3_/ZrO_2_ maintain a lower valence state regardless of rich/stoichiometric/dilute conditions. On the contrary, the worst additive because the strong metal‐support interactions between Rh and non‐stoichiometric oxide Pr_6_O_11_ make Rh more susceptible to oxidation and thus deactivation in oxidizing/stoichiometric atmospheres. They further demonstrated that Rh exhibited a higher ratio of Rh^0^/Rh^3+^ on the Y‐doped ZrO_2_, whereas Pr‐doped ZrO_2_ displayed the lowest ratio, as confirmed by X‐ray absorption near edge structure (XANES) and X‐ray Photoelectron Spectroscopy (XPS). Under dynamic conditions, Rh/Y/ZrO_2_ enhances the T_50_ value of NO by about 60°C compared to Rh/Pr/ZrO_2_, and Y doping can also enhance the catalytic ability under stable conditions. Wang and his co‐workers prepared neodymium‐doped cerium‐zirconium solid solution (CeO_2_‐ZrO_2_‐Nd_2_O_3_) composite oxides using a co‐precipitation method combined with a simple mechanical mixing process (Figure 2c).^[32]^ The addition of trialkyl amine (N235) can effectively maintain higher specific surface area and porosity with lower Ce^3+^ content. It was confirmed by hydrogen temperature‐programmed reduction (H_2_‐TPR) that Rh has a lower valence state when loaded onto CeO_2_‐ZrO_2_‐Nd_2_O_3_ support compared to Rh/ZrO_2_ and still exhibits more metallic Rh after aging. In three‐way catalysts, all Rh/CeO_2_‐ZrO_2_‐Nd_2_O_3_ materials synthesized with the participation of N235 exhibit good propane elimination capability due to their high thermal stability, including large surface area, large pore volume, structural stability, high OSC, and superior reducibility. Compared to the catalyst synthesized without N325, Rh/ZrO_2_/N235 showed a 30°C decrease in T_50_ values for CO and NO and a 96°C decrease in T_50_ value for HC.

**FIGURE 3:**
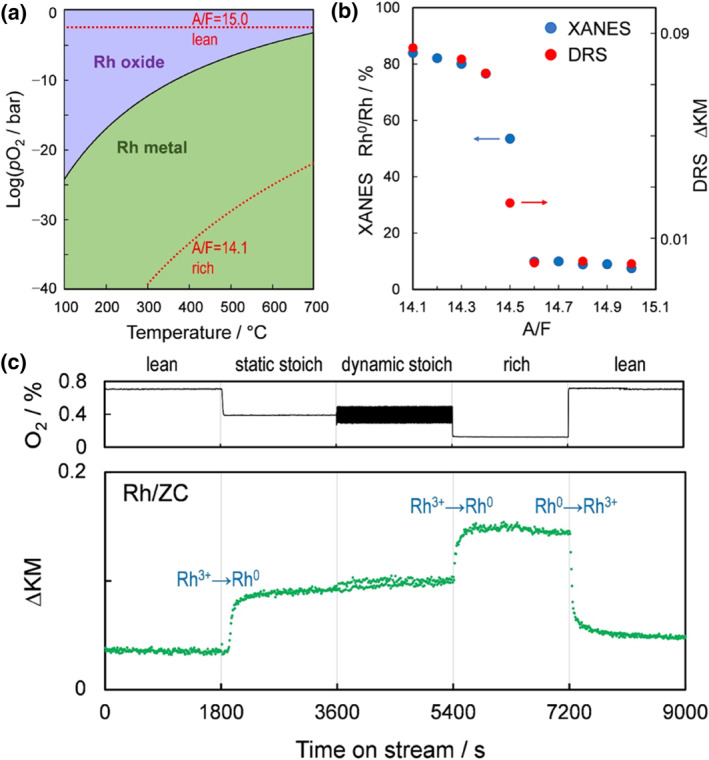
(a) Schematic of phase relationships in the partial oxygen pressure versus temperature plots of the Rh. (b) Kubelka–Munk intensity (ΔKM) and Rh oxidation state as functions of A/F. (c) Kubelka–Munk intensity (ΔKM) at 450 nm for Rh/ZC at 450°C under four simulated gas feeds equivalent.^[35]^ Reproduced with permission.^[35]^ Copyright 2023, Wiley‐VCH GmbH.

A challenge in the application of three‐way catalysts is the transient and non‐stationary fluctuations of the exhaust gas components and temperatures under operating conditions, which are caused by the perturbations between rich and poor fuels under actual combustion conditions, expressed as air‐fuel ratios above and below the stoichiometric point (A/F), or redox ratio (O/R also called λ), respectively.[^33^, ^34^] Such perturbations affect the oxidation state of the platinum group metals and thus the catalytic activity. In order to fundamentally understand the behaviors of Rh at different air‐fuel ratios, Fujiwara and his co‐workers studied the phase diagram of Rh/CeZrO_4_ in different oxygen partial pressures (Figure 3a).^[35]^ They observed the change in Rh species by time‐resolved in situ UV‐vis DRS at A/F from A/F = 15.0 (lean) to 14.1 (rich) and then transformed it to A/F = 15.0 (lean) (Figure 3b). They found that the Rh oxidation state fluctuates synchronously with the A/F uptake, but the oscillation of the Rh/CeZrO_4_ amplitude is smaller than that of Rh/ZrO_2_ (Figure 3c). This is due to the strong deoxygenation ability of cerium‐zirconium solid solution that can avoid the oxidation of the active metal Rh. Thus, the application of ceria‐based supports in three‐way catalysts can accommodate a wide range of air‐fuel ratios due to their oxygen storage capacity and attenuate the oxidative deactivation of Rh in three‐way catalysts. In a high‐temperature oxidizing atmosphere, the strong metal‐support interaction of cerium‐zirconium solid solution with Rh enables Rh to enter the bulk phase. Chinzei has reported that lowering the specific surface area of the supports can effectively retain the metallicity of Rh but sacrifice the OSC.^[36]^ Pyrochlore‐phase cerium‐zirconium solid solutions are derived from reduction treatments at high temperature. Despite the severe sintering of the pyrochlore‐phase, its OSC utilization is highly desirable, allowing Rh to effectively resist high‐temperature aging.

### 2.2 Alumina‐based supports

Commercial porous alumina (γ‐Al_2_O_3_) is often used as a three‐way catalyst support due to its high specific surface area. However, Rh/γ‐Al_2_O_3_ catalysts are easily deactivated at high temperatures under lean fuel conditions.[^37^, ^38^] Therefore, an effective strategy is constructing composite support, where redox supports such as cerium oxide and titanium oxide loaded on alumina are beneficial in separating Rh from alumina and maintaining the high surface area.[^39^, ^40^] Getsoian and his co‐workers loaded Rh onto alumina coated with TiO_2_ or ZrO_2_ by solution atomic layer deposition.^[40]^ These materials exhibited significantly improved low‐temperature conversion compared to modern three‐way catalysts. Doping Ti onto the alumina support improved the dispersion of noble metals and effectively inhibited sintering. Compared to commercial three‐way catalysts, the T_90_ values of Rh/TiO_2_/Al_2_O_3_ for CO, NO, and HC decreased by 59, 115, and 85°C, respectively. Jeong and his co‐workers prepared noble metal (Pt, Pd, and Rh) ensemble catalysts (ESCs) with 100% dispersion by controlling the evaporation temperature of wet impregnation.^[41]^ The noble metals in this catalyst are present as tetrahedral clusters on cerium oxide nano‐islands which are located on the surface of pre‐reduced alumina and are anchored to Al^3+^_penta_ sites. They found lower valence and higher Ce^3+^ content on the metal surface of ESCs compared to single‐atom catalysts (SACs). When ESCs and SACs were used for the three‐way catalysts, ESCs showed good low‐temperature activity for both the oxidation of CO, propylene, and propane and the reduction of NO. For CO and NO, the T_50_ values decreased by 80°C and 90°C, respectively. For HC, ESCs achieved full conversion at 200°C, and SAC left more than half of the HC substrate unconverted even at 400°C due to competitive adsorption.

**FIGURE 4:**
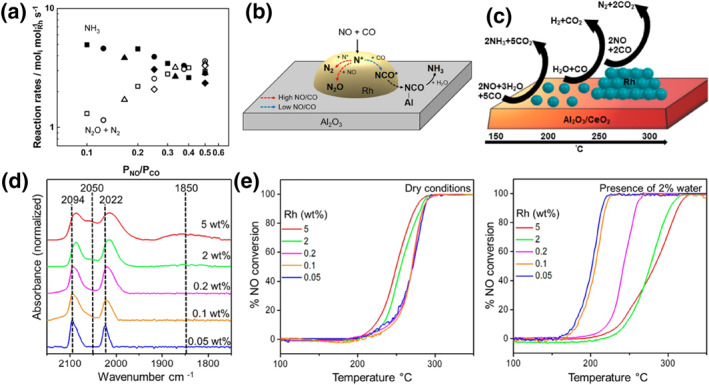
(a) Reaction rates along with P_NO_/P_CO_. (b) NO reduction pathways on Rh/Al_2_O_3_ in the presence of NO/CO/H_2_O mixtures.^[42]^ Reproduced with permission.^[42]^ Copyright 2021, Elsevier. (c) Depiction of product formation during NO reduction over different Rh structures on oxide supports. (d) CO probe molecule FTIR spectra collected for Rh/γ‐ Al_2_O_3_. (e) NO conversion in dry conditions and wet conditions.^[43]^ Reproduced with permission.^[43]^ Copyright 2020, The American Chemical Society.

Yoo and his co‐workers prepared 0.2% Rh/Al_2_O_3_ by wet impregnation.^[42]^ They revealed the selectivity of different NO and CO partial pressures for the products N_2_ and N_2_O, and the NH_3_ selectivity in the presence of water conditions was also explored (Figure 4a). P_NO_/P_CO_ is a descriptor of the reaction rate of nitrogen‐containing products: low CO and high NO pressures favored the formation of N_2_O and N_2_, and high CO and low NO pressures favored the formation of NH_3_. They offered a pathway for the reduction of NO by CO in the presence of water (Figure 4b): (i) under the conditions of higher NO partial pressure, N* atoms dissociated from NO on the Rh surface react with NO to form N_2_O or N_2_; (ii) under the condition of higher CO partial pressure, N* atoms react with CO to form NCO*. The NCO* migrates to the Al_2_O_3_ surface and hydrolyzes with H_2_O adsorbed by the support to produce NH_3_ and CO_2_. Asokan and his co‐workers investigated the structure‐function relationship of Rh sites on alumina and cerium oxide supports (Figure 4c).^[43]^ They prepared clusters and single‐atom Rh/Al_2_O_3_ by controlling the content of Rh precursors. They impregnated Rh precursors onto the Al_2_O_3_ supports and then the mixtures were calcinated at 350°C in air. They confirmed the presence of Rh species are single atoms when the loading amount is below 0.2% and clusters when the loading amount is above 2% by using Fourier Transform infrared spectroscopy (FTIR) (Figure 4d). These catalysts exhibited different catalytic activities under different reaction gas conditions. Under dry conditions, Rh/Al_2_O_3_ with different contents exhibited similar catalytic activities, during which a higher percentage of Rh clusters showed better low‐temperature activities. In the presence of water, the T_50_ value of NO from Rh clusters decreased by only about 10°C. Unexpectedly, the T_50_ value of low‐content Rh exhibited an enhancement of about 60°C, which was attributed to the altered kinetics of NO reduction (Figure 4e). The ammonia formed during the light‐off process adsorbs to the intrinsic acid sites of the alumina supports and thus there is a delay in the formation of NH_3_ during the NO reduction process. When Rh species are present as clusters on alumina, it can significantly minimize the generation of the byproduct NH_3_. The high specific surface area nature of alumina makes it widely used for three‐way catalysts.^[44]^ Moreover, the alumina supports can prevent the sintering of the active center and enhance NO conversion by adsorbing CO and generating NCO* intermediates.

### 2.3 Metal thin film 2D supports

**FIGURE 5:**
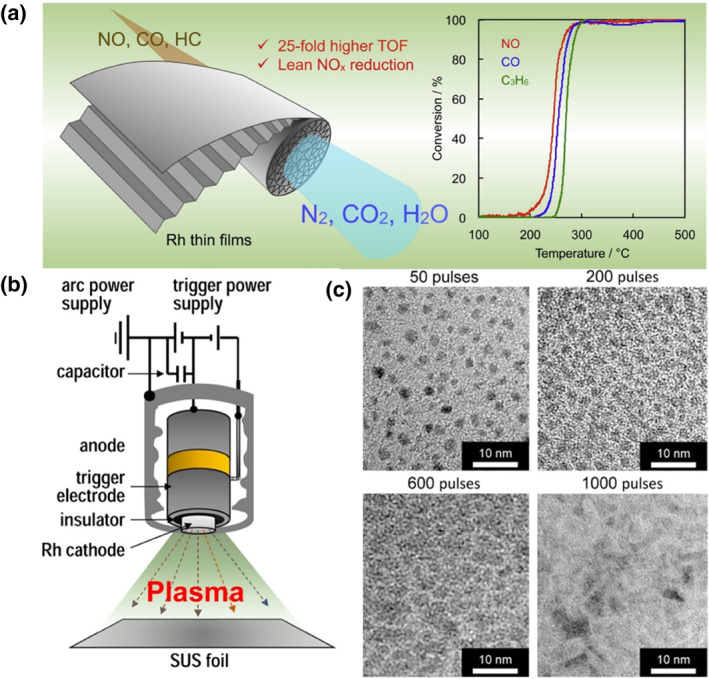
(a) Schematic drawing of Rh thin films.^[48]^ Reproduced with permission.^[48]^ Copyright 2019, Elsevier. (b) Schematic of the pulsed AP deposition of Rh onto an SUS foil. (c) Transmission Electron Microscope (TEM) images of Rh deposited with different numbers of AP pulses.^[49]^ Reproduced with permission.^[49]^ Copyright 2016, Springer Nature.

Commercial three‐way catalysts are usually based on cordierite, which has a honeycomb structure with excellent thermal, chemical, and mechanical properties. Advances in technology have led to an increased focus on metal substrates made of tens of microns of Fe‐Cr‐Al foils, which have higher cell densities and open frontal areas.[^45^, ^46^] With more potential applications, many commercial three‐way catalysts have introduced metal foils. Such substrates are typically loaded with Rh by pulsed arc plasma (AP) deposition.^[47]^ Yoshida and his co‐workers loaded 7 nm thick Rh films on Fe‐Cr‐Al metal foils (Rh/SUS) (Figure 5a).^[48]^ Most of the metallic Rh on the surface of Rh/SUS can retain its low chemical valence. While the metal Rh formed on zirconium dioxide was readily oxidized to the less reactive Rh_2_O_3_ due to the oxidized Rh being more thermodynamically stable. Thus, the antioxidant property of metal Rh was considered as a possible reason for the increased NO conversion efficiency under lean conditions. The T_50_ values of CO, NO, and HC for Rh/SUS at 7 nm thickness are 300, 285, and 340°C, respectively. Misumi and his co‐workers deposited Rh sites of different sizes on Fe‐Cr‐Al thin films using the pulsed cathodic arc‐plasma (AP) technique (Figure 5b).^[49]^ As the pulse intensity was increased from 50 to 600, Rh nanoparticles aggregated and grew. Rh showed a two‐dimensional thin film overlayer structure when the pulse intensity was 1000 (Figure 5c). The two‐dimensional metal film facilitates a more uniform exposure of the crystal surface by enhancing the presence of metallic states. The TOF was increased by 80 times compared with Rh/ZrO_2_/cordierite honeycombs prepared by conventional wet impregnation and slurry coating methods. Since Rh is mostly exposed to the reaction gas with (110) crystalline surface in this preparation method, Rh with a lower coordination number is more active in the dissociation of NO. The T_50_ values of CO, NO, and HC for Rh/foils at 3 nm thickness are 250, 240, and 270°C, respectively. Coating Rh thin film onto metallic film support can provide more Rh sites with a metallic state.

### 2.4 Other supports

**FIGURE 6:**
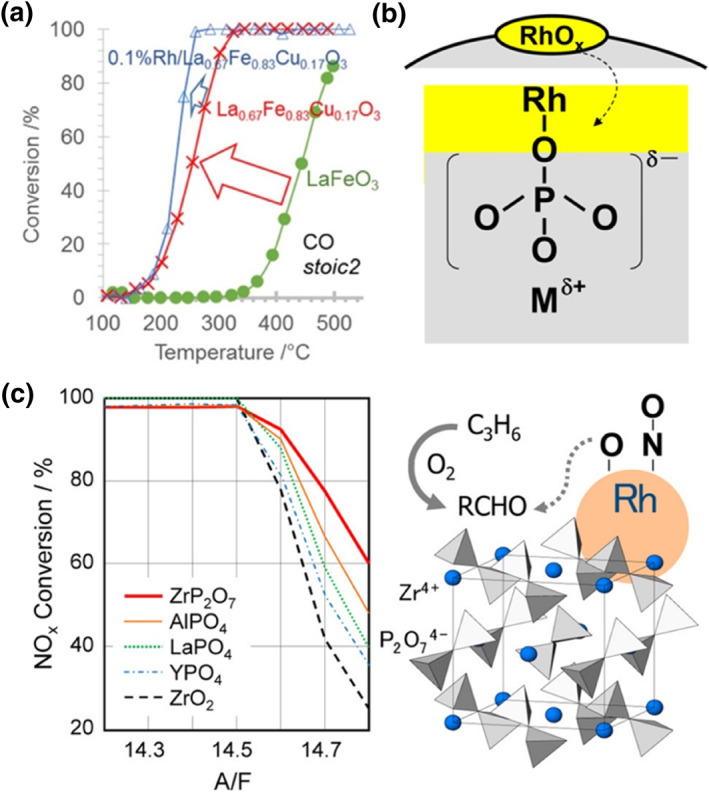
(a) Temperature‐Programmed conversion curves of Rh doped La_1‐y_FeO_3_.^[54]^ Reproduced with permission.^[54]^ Copyright 2018, Elsevier. (b) Schematic drawing of Rh/metal phosphates.^[55]^ Reproduced with permission.^[55]^ Copyright 2016, Wiley‐VCH GmbH. (c) Schematic drawing of Rh/ZrP_2_O_7_.^[56]^ Reproduced with permission.^[56]^ Copyright 2015, The American Chemical Society.

With the development of the synthetic method, and the exploration of new materials gradually entering people's attention, more and more oxides have been used in three‐way catalysts with satisfactory performance. Numerous studies have demonstrated the potential applications of ZnO as a support, such as methane steam reforming, CO oxidation, and propane oxidation.[^50^, ^51^, ^52^] Emiroglu used a hydrothermal method to grow ordered ZnO on cordierite and then impregnated Rh to synthesize Rh‐loaded ZnO.^[52]^ They found that ZnO can easily grow on different supports in the form of ordered arrays. The ordered structure allows a more open front area (OFA), which is beneficial to increase the dispersion of Rh for contacting with the reaction substrates. In addition, the thickness of the ZnO nanowires in the overall cordierite coating is much less than that of the conventional support coating. As a result, there will be reduced exhaust pressure drop and engine power loss in vehicle applications. For Rh/ZnO, NO reacts preferentially with CO below 300°C, and as the temperature increases to 350°C, O_2_ competes with NO to oxidize CO resulting in a weakening of NO elimination. When the temperature is further increased to above 400°C, HC begins to activate NO for abatement. Perovskite‐based materials have been shown to be good redox supports due to their unique ABO_3_ structure, where the A‐site and B‐site can accommodate a wide range of atoms, and the redox capacity can be regulated by adjusting the coordination number and surface vacancies.[^53^, ^54^] Schön and his co‐workers used an impregnation method to load Rh onto La_0.67_Fe_0.87_Cu_0.13_O_3_ perovskite‐based supports (Figure 6a).^[54]^ To regulate the oxidation state of the perovskite surface and to adjust the stoichiometries of La, La^3+^ was partially replaced by Cu^2+^. This low valence elemental substitution leads to a valence change in the B‐site of perovskite and the creation of new lattice oxygen vacancies. They confirmed by H_2_‐TPR that the surface of the Cu‐substituted LaFeO_3_ structure is more reducing, which is conducive to stabilizing the metallic state of the Rh active center. The partial substitution of Cu also increases the number of anionic vacancies on the perovskite surface, which promotes the adsorption and dissociation of NO. Rh/La_0.67_Fe_0.87_Cu_0.13_O_3_ showed similar performance compared to commercial three‐way catalysts with T_50_ values of 247°C, 260°C, and 397°C for CO, NO, and HC, respectively. ZnO can grow on different supports in an ordered structure with a low coating thickness, thus reducing engine exhaust pressure drop and power loss. As a widely used material, perovskite has abundant tunable surface ionic vacancies, which are favorable for regulating the electronic structure of Rh and thus enhancing the catalytic performance.

Metal phosphates are considered harmful products that poison three‐way catalysts, yet they can anchor Rh nanoparticles at high temperatures (1000°C) when used as supports.[^55^, ^56^, ^57^] Machida and his co‐workers studied the metal‐support interactions between metal‐phosphate and Rh (Figure 6b).^[55]^ RhO_x_ is readily reduced to metal Rh on phosphate supports and exhibits high catalytic activity. Rh‐O‐P has a small amount of covalent bonding composition, which is beneficial for stabilizing the surface of Rh nanoparticles and inhibiting sintering under shock‐redox atmospheres. The introduction of P‐OH on the surface contributed to the activation of propylene, thereby promoting the reduction of NO. Rh/AlPO_4_ showed only about 70°C decrease in performance after aging, while Rh/Al_2_O_3_ showed severe deactivation. Nagao and his co‐workers further investigated the effect of different cations on Rh‐based three‐way catalysts (Figure 6c).^[56]^ Four different metal phosphate supports were prepared by co‐precipitation and then loaded with Rh by impregnation. They found that the support activity followed the following sequence: ZrP_2_O_7_ > AlPO_4_ > LaPO_4_ > YPO_4_ > ZrO_2_. The ZrP_2_O_7_ supports could oxidatively adsorb propylene to aldehydes rather than carboxylic acids during the reaction, which was beneficial to inhibit the competitive adsorption of oxygen and thus facilitate further oxidation. In three‐way catalysts, the presence of phosphorus (P) can lead to catalyst poisoning due to the formation of phosphates. However, when metal phosphates are employed as supports, their strong anchoring effect can enhance the sinter resistance ability of Rh on the surface.

For Rh‐based three‐way catalysts, the main functions of the support are: to provide a high surface area, to extend the air‐fuel ratio working interval, and to inhibit the deactivation of surface‐active metals, especially Rh. The valence state of Rh has a considerable influence on the three‐way catalysts. Isolated single‐atomic sites with the highest oxidation states are not conducive to HC elimination in three‐way catalysts. Making Rh exhibit lower valence and higher metallicity is conducive to improving the activity of three‐way catalysts. Supports influence the electronic structure, metal atom mobility, metal size, etc. of Rh through metal‐support interactions. For most supports such as ceria‐based alumina‐based and phosphates, stronger metal‐support interactions tend to provide Rh a higher oxidation state and more stable binding energy, which are detrimental to three‐way catalysis.^[58]^ Appropriate metal‐support interaction can enhance the activity or stability. How to select a suitable support to regulate metal‐support interactions is very important. Therefore, optimizing the adsorption of metals with reactive molecules and the subsequent low‐temperature activity and high‐temperature stability need to be studied.

## 3 ACTIVE CENTER CONSTRUCTION STRATEGIES IN RH‐BASED CATALYSTS

In the previous section, the interactions between metal and support were investigated to find several suitable supports targeted to enhance the activity of three‐way catalysts. However, a single active site cannot satisfy the simultaneous enhancement of sintering resistance and low‐temperature catalytic activity.^[28]^ Therefore, the intermetallic synergy is modulated by introducing lower‐cost metals to create synergistic effects with Rh to reduce costs and improve catalytic capabilities.^[11]^ This chapter summarizes the breakthroughs of three‐way catalysts in bimetallic as well as trimetallic synergism in recent years.

### 3.1 Metal alloy active center

**FIGURE 7:**
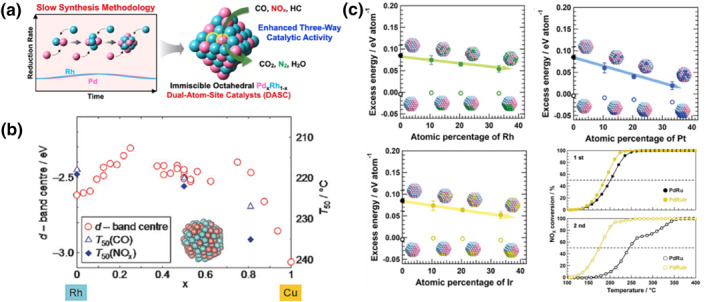
(a) Schematic drawing of Pd_x_Rh_1‐x_ dual‐atom‐site catalysts.^[62]^ Reproduced with permission.^[62]^ Copyright 2022, Wiley‐VCH GmbH. (b) Schematic of Rh‐Cu alloy.^[63]^ Reproduced with permission.^[63]^ Copyright 2017, Wiley‐VCH GmbH. (c) The composition dependence of the excess energies of PdRuM NP models at 0 K and NO_x_ reduction activity.^[64]^ Reproduced with permission.^[64]^ Copyright 2021, Wiley‐VCH GmbH.

One of the most attractive advantages of solid solution alloys is the ability to modulate the surface electronic structure by controlling the composition, thereby affecting their physicochemical properties.^[59]^ The alloys can acquire new unique properties or enhance synergies by mixing two metals. However, the synthesis of alloys in solid solutions still faces great challenges, especially for immiscible alloys whose constituent elements are not homogeneously mixed in the bulk phase.[^60^, ^61^, ^62^] The alloys are often thermodynamically non‐equilibrium phases at room temperature and are prone to surface segregation at elevated temperatures, which disrupts the homogeneous alloy structure and results in reduced catalytic activity.^[56]^ Tan and co‐workers developed a slow synthetic method for immiscible Pd and Rh alloy systems to obtain the entire range of Pd_x_Rh_1‐x_ (x = 0–0.5) (Figure 7a).^[62]^ They maintained a very low noble metal solution concentration by prolonging the addition time of the mixed precursor solution, and the Pd_x_Rh_1‐x_ alloys at a range of concentration gradients showed an octahedral morphology. In this case, the coordination of the noble metals in the precursor solution determines the morphology of the final products, with the K_2_PdCl_4_ and Na_3_RhCl_6_ precursors having higher as‐reduced atomic energy levels and tending to aggregate into octahedral clusters, while the Pd(NO_3_)_2_ and RhCl_3_ precursors tending to form spherical nanoparticles. Among these PdRh alloys with different ratios Pd_0.3_Rh_0.7_ exhibits better catalytic activity than pure Rh because the asymmetric dissociation splitting of NO molecules by PdRh is faster than the symmetric dissociation of a single Rh site. Compared to pure Rh, the T_50_ values of CO, NO, and HC of Pd_0.3_Rh_0.7_ decreased by 10, 8, and 10°C, respectively. Furthermore, considering Pd is nearly six times less expensive than Rh, the PdRh alloy demonstrates superior economic viability compared to pure Rh nanoparticles.

To reduce the utilization of Rh in three‐way catalysts, Komatsu and his co‐workers obtained the density of states (DOS) of Rh_1‐x_Cu_x_ alloys with different contents by density functional theory (DFT) calculations and speculated that the catalytic activity was correlated with the DOS (Figure 7b).^[63]^ They then obtained Rh_1‐x_Cu_x_ (0 < x < 0.81) alloy NPs by co‐reduction where potassium tert‐butoxide was added to enhance the reduction property, and this synthesis method provides an effective synthetic route for not only solid solution alloys of bulk immiscible elements but also alloys consisting of oxidizable elements. They found that Rh_0.5_Cu_0.5_ was stable at 750°C without obvious phase separation, and the catalytic activity of Rh_0.5_Cu_0.5_ did not decrease significantly compared with pure Rh, consistent with DOS calculations. The T_50_ values for CO and NO for Rh_0.5_Cu_0.5_ are 220 and 222°C, respectively, while the T_50_ value for HC conversion is significantly higher than that of CO and NO, which may be due to the different reaction mechanisms for HC elimination compared to CO and NO. To further minimize the Rh content, Kusada and co‐workers exploited the conformational entropy effect to achieve Pt, Rh, and Ir miscibility in PdRu solid solutions for three‐way catalysts. Ternary alloys are produced by the triethylene glycol‐assisted co‐reduction method (Figure 7c).^[64]^ They confirmed by energy dispersive x‐ray spectroscopy and synchrotron x‐ray diffraction that the three elements are homogeneously mixed and have a single fcc structure. Compared with binary PdRu catalysts, ternary alloy catalysts not only have significant stability but also have good TWC reactivity. The T_50_ values of PdRuRh catalysts for CO, NO, and HC are 210, 195, and 255°C, respectively. To further illustrate the effect of conformational entropy on alloys, they introduced the concept of excess energy (ɛ_excess_) which is defined as Equation (9). For PdRuM (N = N_Pd_ + N_Ru_ + N_M_ = 201), N_Pd_, N_Ru_, and N_M_ are the numbers of Pd, Ru, and M atoms in PdRu(M) NPs, respectively. When M is Pt, Rh, or Ir, the addition of the third metal can reduce the excess energy of the PdRuM, thus stabilizing the ternary alloy and making it difficult to phase segregation; when M is Ag or Au, the excess energy of the ternary alloy will increase. Given the Gibbs free energy (G = H–TS), the ternary alloy exhibits thermodynamic instability at Taman temperature, and a higher conformational entropy leads to larger TS values and more negative G values. In the PdRuM system, Pt, Rh, and Ir exhibit greater conformational entropy, thereby stabilizing the ternary alloy at Taman temperature. However, Ag and Au do not contribute to this stabilization effect. Therefore, the ternary alloy PdRuRh has a high stability. It is worth noting that PdRuIr not only has excellent catalytic properties but also lower raw material costs.

(9) $$\varepsilon_{excess}=\frac{1}{N}\left(\varepsilon_{PdRuM}-\frac{N_{Pd}}{N}\varepsilon_{Pd}-\frac{N_{Ru}}{N}\varepsilon_{Ru}-\frac{N_{M}}{N}\varepsilon_{M}\right)$$

### 3.2 Phase separation metallic actives center

**FIGURE 8:**
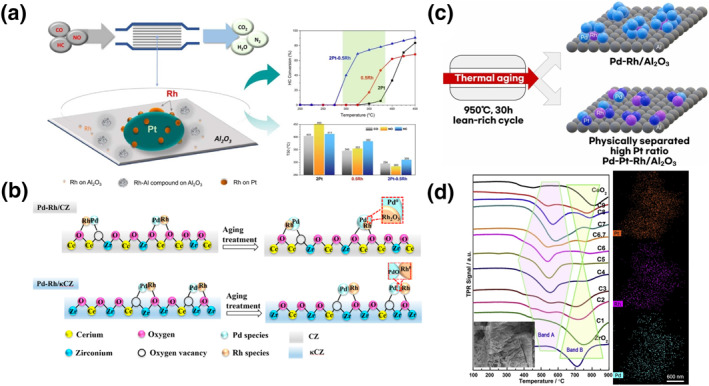
(a) Schematic drawing of Pt‐Rh synergy for three‐way catalysis.^[66]^ Reproduced with permission.^[66]^ Copyright 2023, Elsevier. (b) Schematics describing the thermal evolution of noble metal species for Pd−Rh bimetallic catalysts.^[67]^ Reproduced with permission.^[67]^ Copyright 2022, The American Chemical Society. (c) Schematic of Pd–Pt–Rh trimetallic catalysts.^[68]^ Reproduced with permission.^[68]^ Copyright 2023, Elsevier. (d) Schematic of OSC and surface area and the relationship of CZ solid solutions in different compositions.^[69]^ Reproduced with permission.^[69]^ Copyright 2019, Elsevier.

For bimetallic or polymetallic catalytic materials, figuring out how to make them into alloys is certainly an interesting route, but due to the difficulty of their synthesis and room temperature thermodynamic instability, many studies have been carried out from phase‐separated metallic materials in which Pt, Rh and Pd are immiscible with each other.[^65^, ^66^, ^67^, ^68^, ^69^] Li and his co‐workers investigated the synergistic effect of PtRh‐loaded alumina with different Pt: Rh ratios in three‐way catalysts. The catalysts were prepared by impregnating different contents of Pt and Rh on alumina and then calcined at 550°C for 2h subsequently (Figure 8a).^[66]^ Among them, the 2Pt0.5Rh material contained three forms of Rh presence on the aged surface: small Rh nanoparticles on island‐shaped Pt particles, Rh nanoparticles on supports, and Rh single‐atoms on supports. They found that a synergistic effect could only be achieved if Pt and Rh were in contact with each other. The Rh site could more easily remove adsorbed oxygen atoms by electron transfer from Pt, resulting in a higher activity at low temperature. On the other hand, the second benefit of this interaction may come from the change in the physical and electronic states of Pt, which enhances the sintering resistance of Pt nanoparticles. The catalytic activity of 2Pt0.5Rh was superior to that of 0.5Rh, and 0.5Rh was superior to that of 2Pt. Compared to 0.5Rh, 2Pt0.5Rh achieved a decreased T_50_ values for CO, NO, and HC by 51, 70, and 73°C, respectively. Yin et al. studied the three‐way performance of ceria‐zirconia solid solutions with different phases. The catalysts were prepared by impregnating the Pd and Rh precursors on conventional or pyrochlore phases ceria‐zirconia and calcined at 600°C for 3 h (Figure 8b). They found that in the conventional phase ceria‐zirconia solid solution, Rh species are easily oxidized due to the strong metal‐support interactions between Rh and the ceria‐zirconia solid solution, and Pd sintered to agglomerate easily due to the weak interactions between Pd and the support. The two different interactions lead to the Pd segregation on the surface and reduce the catalytic activity when alloying Rh and Pd. When pyrochlore‐phase ceria‐zirconia solid solutions were used, Rh and Pd metal‐support interactions were reduced and enhanced, respectively, and Pd/Rh tended to maintain good three‐way catalysts performance states, including better PdO stability and higher concentration of Pd/Rh active states. Accordingly, the three‐way catalyst performance of the Pd‐Rh/κCZ catalysts was enhanced, and T_90_ values for NO and HC were reduced by 10 and 15°C, respectively. Many researchers have demonstrated that bimetallic and trimetallic catalysts with optimal metal ratios are more active than monometallic catalysts.

Woo and his co‐workers prepared Pt‐Rh‐Pd trimetallic catalysts by using co‐impregnation (PdPtRh‐co), sequential impregnation (PdPtRh‐seq), and physical mixing methods (PdPtRh‐pm) (Figure 8c).^[68]^ They demonstrated by XPS that the Rh of the PdPtRh‐seq sample has a lower valence state compared to the other samples thus favoring NO reduction. They also demonstrated by H_2_‐TPR that the PtO_x_ species of PdPtRh‐seq is easier to be reduced, thus showing a better ability to oxidize CO and HC. After aging the above catalysts, they found that the transformation of Pt species into PtO_2_ under oxidizing conditions was the main cause of deactivation. The PdPtRh catalyst synthesized by sequential impregnation inhibited the formation of PtO_2_ because of the preferential impregnation of Pt. The PdPtRh‐pm catalyst consisted of a mixture of PdPt‐seq and RhPt‐seq. They further found that sintering of PdRh alloys could be avoided effectively when Pd and Rh were physically separated, so compared to the PdPtRh‐seq sample, PdPtRh‐pm inhibited both oxidative deactivation of Pt species and sintering of RhPd alloy. Ouyang and co‐workers studied the effect of different Ce: Zr ratios in PtRhPd‐loaded ceria‐zirconia solid solutions for three‐way catalysis. PtRhPd trimetallic catalysts were prepared by impregnation and calcination at 400°C (Figure 8d).^[69]^ They controlled cerium‐zirconium solid solutions with different cerium‐zirconium ratios and impregnated RhPd and Pt consecutively. They used TEM to observe the morphology of the samples and high‐angle annular dark‐field (HAADF) mapping to demonstrate the uniform loading of the three metals (Pt, Rh, and Pd) on the surface. They found that the ceria‐zirconium ratios of the solid solutions show volcano‐type curves in specific surface area, oxygen storage capacity, and three‐way catalysis performance as well. When the cerium‐zirconium ratio is 1:1, supports have maximum oxygen storage capacity and porosity, which is favorable for the dispersion of noble metals and mass transfer of gases (CO, NO_x_, and hydrocarbons). The T_90_ values of CO, NO_x_, and HC for the cerium‐zirconium ratio 1:1 sample were 284, 291, and 323°C, respectively. The metal‐support interactions of conventional phase ceria‐zirconia solid solutions on Rh are different. Strong metal‐support interactions of Rh lead to a higher oxidation state of Rh. Pyrochlore phase ceria‐zirconia solid solutions can decrease the metal‐support interactions of Rh, thus decreasing the high valence state. The different cerium‐zirconium ratios of conventional ceria‐zirconium solid solutions also affect the three‐way catalyst performance by OCS.

### 3.3 Physical separation active center

**FIGURE 9:**
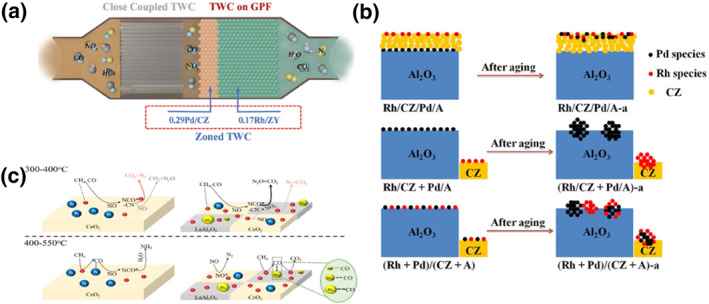
(a) Schematic illustration of the zoned Rh‐ and Pd‐based three‐way catalysts.^[72]^ Reproduced with permission.^[72]^ Copyright 2023, Elsevier. (b) Schematics describing the hydrothermal evolution of noble metal species.^[73]^ Reproduced with permission.^[73]^ Copyright 2019, Wiley‐VCH GmbH. (c) Schematic of the formation mechanism of N_2_O and NH_3_ on the PtRh three‐way catalyst.^[74]^ Reproduced with permission.^[74]^ Copyright 2023, Elsevier.

As mentioned above, direct contact between the two metals in an oxidizing atmosphere at high temperatures tends to lead to sintering, segregation, resulting in reduced activity. Physically separating the two sinter‐prone metals would be a fundamental solution to the sintering problem, as opposed to regulating metal‐support interactions or metal synergies to inhibit sintering.^[70]^ Some commercial catalysts utilize zoned or layered catalysts, where the coating and active metal of the zoned catalyst varies along the length of the monomer, and layered catalysts sequentially deposit multiple layers of active metal on the monomer to inhibit alloy formation.[^71^, ^72^, ^73^, ^74^, ^75^] Xin and his co‐workers investigated the stability of Rh and Pd in three‐way catalysts. They prepared zoned RhPd three‐way catalysts via the ethylene glycol reduction method. The Pd and Rh nanoparticles were subsequently loaded onto the supports (Figure 9a).^[72]^ They loaded 0.17% Rh onto Y‐modified ZrO_2_, the highly dispersed Rh species had excellent performance in NO reduction, and the Y‐modified ZrO_2_ strengthened the metal‐support interactions of Rh, thereby inhibiting the sintering of Rh and the partitioning of Y. The catalysts were characterized by a high degree of sintering and partitioning. They also loaded 0.29% Pd onto cerium‐zirconium solid solutions, which provided excellent CO and HC reduction due to the combined effect of support‐rich reactive oxygen species and Pd species. When Pd and Rh are zoned in a ratio of 1:4, the advantages of Pd and Rh are combined and PdRh sintering is avoided. The zoned catalyst exhibits optimized catalytic activity with the widest operating temperature range, and the T_50_ values of CO, NO and HC are 210, 235 and 250°C respectively. Lan and his co‐workers explored the promotion effects of loading position and order of Rh and Pd on different supports in three‐way catalysis. Rh/CZ/Pd/A was prepared via a modified impregnation method. The positions of Rh and Pd were controlled by adjusting the impregnation sequence (Figure 9b).^[73]^ When Rh and Pd were separately impregnated onto two different supports, both Rh and Pd showed severe aggregation and generated inactive oxidation states, when Rh and Pd were co‐impregnated on two different physically mixed supports, Rh and Pd interacted with each other the most, which was detrimental to the three‐way catalysts, and the large alloy particles were easily formed during aging. During hydrothermal aging, the Pd species in the inner layer of Rh/CZ/Pd/A moved outward to the CZ layer, and the CZ layer on Al_2_O_3_ maintained a high specific surface area, which enhanced metal‐support interactions of Pd, and hydrothermal stability of Rh. Compared to the other two catalysts, the T_50_ values of Rh/CZ/Pd/A exhibited a decrease in CO and NO of about 30°C.

Zhang and his co‐workers investigated the promotion mechanism of separated Pt and Rh active centers in three‐way catalysts. They prepared Pt/Ce‐Rh/LA by impregnating Pt and Rh on cerium oxide and La‐modified alumina, respectively (Figure 9c).^[74]^ They discovered the formation mechanism of N_2_O and NH_3_ on PtRh physically separated catalysts. There are two possible mechanisms for N_2_O: surface CN species competing with NO_2_ or O_2_ to form N_2_O, and surface NCO* species reacting with surface nitrate. Both reaction pathways are affected by the concentration of surface adsorbed oxygen (O_ads_), and high concentrations of O_ads_ will accelerate the low‐temperature oxidation of NO. The formation of NH_3_ comes from the adsorption of HC species on the Rh surface and then react with NO. The high concentration of O_ads_ on the surface promotes the oxidation of HC on the Rh surface, thereby inhibiting the reaction of NO with HC and reducing the formation of NH_3_. The T_50_ values of Pt/Ce‐Rh/LA for NO and HC were 380°C and 370°C, respectively. Lee and his co‐workers investigated the kinetics of the (111) surfaces of Pt, Rh, and Pd in three‐way catalyst reactions by DFT calculations.^[75]^ They found that Pt and Pd exhibited similar kinetic behaviors, with H_2_ playing an important role in assisting NO dissociation and removing O* from the Pt surface. For Rh, the catalysts exhibited high NO reduction activity under fuel‐rich conditions, and they calculated that the catalysts of Pt and Rh physical mixtures had the most excellent NO reduction activity. Synergistic interactions between Rh and different metal sites can enhance the catalytic activity of Rh‐based catalysts by modulating the electronic structure, density of states, and metal‐support interactions of Rh. Physical mixing of Rh with different metals can fundamentally address the sintering of Rh with another species to obtain high stability. The elucidation of metal synergies in three‐way catalysts is expected to enable the design of high‐performance catalysts.

## 4 MECHANISM AND SPECIES BEHAVIOR DURING THREE‐WAY REACTION

To optimize the performance of three‐way catalysts, it is incomplete to understand only the metal‐support interactions and the electronic structure of the active sites. It is imperative to understand the adsorption effects of active sites for reacting molecules at the molecular level.^[9]^ Three‐way catalysts effectively catalyze the degradation of CO, NO, HC, and multiple gas components imply the multiple reaction pathways.^[1]^ Therefore, a clear knowledge of the basic steps of these reactions and the corresponding kinetic mechanisms is essential to elucidate the behavior of the reaction system.

### 4.1 Mechanism of Rh‐based three‐way catalysts

**FIGURE 10:**
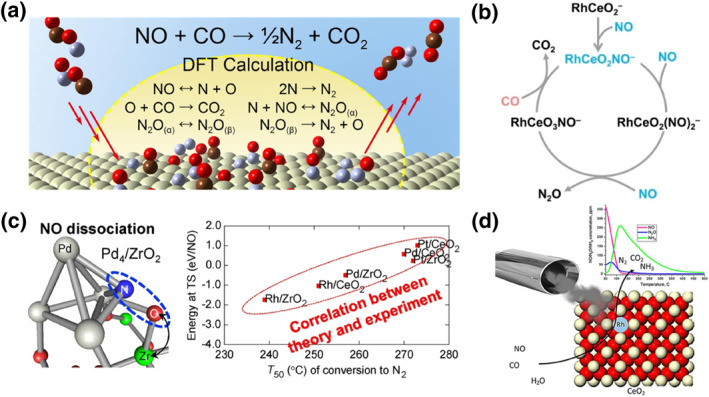
(a) Schematic illustration of the mechanism of NO + CO on Rh.^[77]^ Reproduced with permission.^[77]^ Copyright 2018, Elsevier. (b) Proposed catalytic cycle of NO reduction by CO mediated with NO pre‐adsorbed RhCeO_2_NO.^[78]^ Reproduced with permission.^[78]^ Copyright 2023, Wiley‐VCH GmbH. (c) Schematic of ceria and zirconia supports on NO reduction.^[79]^ Reproduced with permission.^[79]^ Copyright 2019, Elsevier. (d) Schematic illustration of single‐atom rhodium on ceria for NO abatement.^[80]^ Reproduced with permission.^[80]^ Copyright 2021, Wiley‐VCH GmbH.

Three‐way catalysis contains many reactions, and it is difficult to propose reasonable mechanisms and pathways under conditions where so many reactions are carried out simultaneously. Therefore, most mechanistic studies have focused on the CO + NO reaction and the selectivity of NO elimination.^[76]^ The classical reaction pathways of CO and NO on the Rh surface are realized by the following steps: reversible adsorption of CO and NO, dissociation of NO to form atoms N and O, recombination of N atoms to form N_2_, reaction of O with CO to form CO_2_, reaction of N with NO to form N_2_O, and dissociation of N_2_O to form N_2_ and atoms O.^[24]^ Tan and his colleagues used DFT to calculate the kinetics of the three‐way catalysis for different crystal faces of the metal Rh (Figure 10a).^[77]^ They found that in three‐way catalysts: (1) the combination of NO with O to produce NO_2_ is not an easy step due to its very high activation energy. NO prefers to dissociate at low coverage or to desorb at high coverage. Therefore, NO_2_ is a negligible by‐product of the three‐way catalytic reaction; (2) Rh (111) is the densest, while Rh (100) is slightly more open. Multiple reactants, as well as intermediates, may share the same Rh atom on the Rh (111) surface, whereas almost every reacting molecule is bound to two Rh atoms on the Rh (100) surface, and this difference results in a more stable transition state and a lower reaction potential on Rh (100). Rh (100) possesses superior selectivity for N_2_ compared to N_2_O; (3) The N_2_ generation step is divided into two parts: the complexation of 2 N atoms and the dissociative desorption with N_2_O as an intermediate. Through DFT calculations, they found that the energy barriers for N_2_O formation are slightly lower than those for the direct combination of 2 N atoms, and thus essentially N_2_O is easier to form. However, this advantage is not determinative, the activation energy of N‐atom recombination receives more influence from the surrounding environment, and the energy barriers for N‐atom recombination in certain environments may be lower than those for N_2_O formation. Chen and his co‐workers investigated the behavior of RhCeO_2_ clusters in the CO + NO reaction using mass spectrometry for cluster reactions, cryogenic photoelectron imaging spectroscopy, and quantum‐chemical calculations (Figure 10b).^[78]^ They found that at least three NO molecules are required for the catalytic cycle of CO + NO to produce N_2_ and emphasized the important role of NO in the reaction, which is not only a regulator but an indispensable promoter of the reaction. Pre‐adsorbed NO recombines the rhodium‐cerium oxide cluster RhCeO_2_ and generates a Ce^3+^ ion in the product RhCeO_2_NO, which is the starting point for triggering and driving NO reduction by CO. The reduction of the other two NO molecules to nitrous oxide occurs only on this newly generated Ce^3+^ ion, and Rh acts like a promoter to regulate the electrons and synergizes with Ce^3+^ to promote the catalytic reaction.

Koga and his co‐workers constructed tetrahedral M_4_ clusters on CeO_2_ and ZrO_2_ (Figure 10c).^[79]^ They calculated and investigated the adsorption kinetics and reduction activity of the metal clusters on NO using DFT. They found that the M_4_ clusters were positively charged on cerium dioxide but nearly neutral on zirconium dioxide and that NO was slightly more negatively charged on M_4_/ZrO_2_ than on M_4_/CeO_2_. The reaction of CO with adsorbed NO did not show a strong support dependence, and in contrast, the dissociation of NO at the metal/oxide interface did. The activity of CO reacting with adsorbed NO increased in the order of Pt < Pd < Rh order of increase. ZrO_2_ is more favorable for the dissociation of NO because Zr in zirconium dioxide carries a more positive charge than Ce in cerium oxide and interacts more strongly with the O‐terminus of NO. Although ZrO_2_ has a slightly better reactivity to NO than CeO_2_, cerium oxide has a better oxygen storage capacity, which is beneficial to adapt to different air‐fuel ratios, so the reasonable regulation of the presence of cerium oxide and zirconium oxide when designing the catalyst supports is beneficial to the design of three‐way catalysts.

Khivantsev and his colleagues investigated the behavior of Rh single‐atoms on cerium oxide by studying the CO + NO reaction (Figure 10d).^[80]^ Subsequently, they revealed the different pathways as well as the selectivity of the CO + NO reaction under dry versus aqueous conditions. Two main pathways are involved in the catalytic behavior under dry conditions, as shown in Equations (10) and (11):

(10) $$CO+NO\;\to \;{CO}_{2}+0.5\;N_{2}$$

(11) $$CO+2NO\;\to \;{CO}_{2}+N_{2}O$$

Rh single‐atom participation in the drying condition CO + NO is shown in Equations (12) and (13):

(12) $${Rh}^{I}+2NO\;\to \;{Rh}^{I}{\left(NO\right)}_{2}\;\to \;{Rh}^{I}{\left(NO\right)}^{\delta +}{\left(NO\right)}^{\delta -}\to \;{Rh}^{III}\left(N_{2}O_{2}\right)$$

(13) $${Rh}^{III}\left(N_{2}O_{2}\right)+CO\;\to \;{Rh}^{III}\left(CO\right)\left(N_{2}O_{2}\right)\to \;{Rh}^{I}+N_{2}O+{CO}_{2}$$

They found that N_2_O began to form at 50°C when water was present in the reaction. To explain this phenomenon, they proposed that the Rh^III^(N_2_O_2_) hyponitrite intermediate is hydrolyzed by water or protons on the surface of cerium oxide, and the resulting hyponitric acid is unstable above room temperature will decompose to produce N_2_O. CO subsequently reacts with the Rh^III^ hydroxo complex to form a carboxyl Rh^III^ complex, which finally dissociates to CO_2_ and water and restores the initial Rh^I^ site, as shown in Equations ((14), (15), (16), (17)):

(14) $${Rh}^{III}\left(N_{2}O_{2}\right)+2H_{2}O\;\to \;{Rh}^{III}{\left(OH\right)}_{2}+H_{2}N_{2}O_{2}$$

(15) $$H_{2}N_{2}O_{2}\;\to \;H_{2}O+N_{2}O$$

(16) $${Rh}^{III}{\left(OH\right)}_{2}+CO\;\to \;{Rh}^{III}\left(CO\right){\left(OH\right)}_{2}\;\to \;{Rh}^{III}\left(COOH\right)\left(OH\right)$$

(17) $${Rh}^{III}\left(COOH\right)\left(OH\right)\;\to \;{Rh}^{I}\left(H\right)\left(OH\right)+{CO}_{2}\;\to \;{Rh}^{I}+H_{2}O+{CO}_{2}$$

Alternatively, CO can be oxidized to carbonate species by nucleophilic attack of OH species, as shown in Equation (18):

(18) $${Rh}^{III}{\left(OH\right)}_{2}+CO\;\to \;{Rh}^{III}\left(H_{2}{CO}_{3}\right)\;\to \;{Rh}^{I}+H_{2}O+{CO}_{2}$$

Alternatively, ammonia can be formed via hydrogen stored on Rh in the water‐gas reaction path, as shown in Equation (19):

(19) $$Rh-OH+CO\;\to \;Rh\left(COOH\right)\;\to \;Rh-H+{\;CO}_{2}$$

They reveal that Rh^I^ single‐atoms dispersed on cerium oxide are highly reactive species for the CO + NO reaction and are tremendously robust. This species is also highly active and selective for NO degradation by wet industrial three‐way catalysts, and can completely degrade NO above 120°C. They have discovered that the Rh‐H species is a key species for NO hydrogenation and that ammonia formation is highly dependent on Rh single‐atom water‐gas shift (WGS) reactivity. This new stable and excellent low‐temperature performance three‐way catalyst with 100% Rh atom economy is very promising. In the three‐way catalysts, the metallic Rh is more active compared to the single‐atom Rh, which has low oxidation activity towards HC. In the CO + NO model reaction, both metallic and single‐atom Rh show good activity, which is due to different reaction mechanisms. When Rh is present as a cluster, it is in the metallic state. NO dissociates on the metal surface to form N_2_ or N_2_O. When the Rh center is present as a single‐atom, Rh simultaneously adsorbs CO, NO, and water. Besides, the support also affects the reaction by adsorbing NO.

### 4.2 Migration and encapsulation of Rh species

**FIGURE 11:**
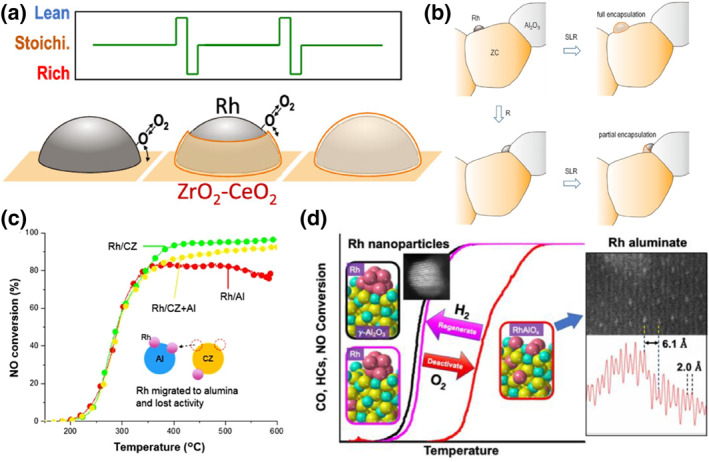
(a) Schematic diagram of the possible Rh encapsulation mode.^[84]^ Reproduced with permission.^[84]^ Copyright 2023, The American Chemical Society. (b) Schematic diagram of possible changes in the supported Rh nanoparticles.^[85]^ Reproduced with permission.^[85]^ Copyright 2023, The American Chemical Society. (c) Schematic of migration of rhodium under air atmosphere.^[86]^ Reproduced with permission.^[86]^ Copyright 2021, Elsevier. (d) Schematic illustration of deactivation of Rh/Al_2_O_3_.^[87]^ Reproduced with permission.^[87]^ Copyright 2022, The American Chemical Society.

Automotive three‐way catalysts experience their worst thermal aging at temperatures close to 1000°C. A major challenge is to improve catalyst stability over longer periods by minimizing the utilization of platinum group metals (PGMs).^[81]^ Rh is susceptible to high‐temperature aging under fluctuating conditions of air or exhaust gas composition in the stoichiometric (S)‐lean (L)‐rich (R) sequence, which produces behaviors such as migration and encapsulation of Rh species, leading to deactivation of catalysts. Under dynamic conditions, the oxidation state and catalytic activity of the Rh nanoparticle surfaces are forced to oscillate accordingly.^[82]^ To reduce these effects, ceria‐zirconia solid solutions with alumina were used as supports. However, the ZC material plays a central role in the rapid uptake/release of oxygen in the interfacial region with the PGM particles, a behavior in which atoms can accelerate decoration or encapsulation by migration.^[83]^ Machida and his co‐workers investigated the thermal deactivation behavior of Rh/ZC three‐way catalysts after engine aging at static S, L, and 1000°C for 40 h (Figure 11a).^[84]^ The most severe catalyst deactivation after aging in the SLR cycle was attributed to the almost complete encapsulation of the support ZC, where a significant portion of the Rh surface was unavailable for chemisorption and catalysis. This phenomenon is strongly temperature‐dependent and is evident at aging temperatures above 900°C. The SLR cycle induces rapid and repetitive release and storage of oxygen, which involves dissociation and recombination of O on Rh, spillover to the CZ, and formation of lattice oxygen vacancies. Remarkably, dynamic interfaces will activate the migration of ZC components with surface energy as the driving force to cover Rh nanoparticles. However, it does not occur when Rh is on an alumina support. They also found that pre‐aging the catalysts with cerium‐zirconium solid solution and alumina as supports under fuel‐rich conditions at 1000°C can greatly alleviate this deactivation mode (Figure 11b).^[85]^ *R* aging promotes Rh nanoparticle migration from the ZC support to the alumina boundary. Due to the interaction with the alumina strong interaction, Rh particles are trapped near the boundary and these Rh nanoparticles are not easily and completely covered by ZC even under SLR conditions. Tomida and his co‐workers synthesized several series of Rh‐loaded CZ and Al_2_O_3_ catalysts by impregnation: Rh/CZ + Al, Rh/Al + CZ, Rh/CZ, and Rh/Al (Figure 11c).^[86]^ They found that when the catalysts were hydrothermally aged at 1000°C, Rh/CZ + Al and Rh/Al + CZ exhibited similar catalytic properties. To explain this phenomenon, they found that both catalysts had the same Rh species after hydrothermal aging by H_2_‐TPR and XPS. Rh was covered on both CZ and alumina surfaces and a large number of RhAlO_x_ species were present.

### 4.3 Formation of RhAlO_x_

Three‐way catalysts are commonly supported by cerium oxide‐based supports and alumina‐based supports. The disadvantage of the excellent oxygen storage and release capability of cerium oxide‐based supports is the encapsulation of noble metals by the supports in the redox cycling atmosphere.[^87^, ^88^, ^89^, ^90^, ^91^] Due to its stability, alumina does not encapsulate noble metals on the surface. However, Rh/Al_2_O_3_ is severely deactivated above 900°C under purely oxidizing conditions.[^87^, ^89^, ^90^] It is in extreme conditions that most of the catalyst aging in real driving conditions occurs, so it is necessary to understand the specific deactivation behavior of Rh on catalysts. The formation of RhO_x_ species on alumina during high‐temperature oxidation conditions has been identified as the primary cause of deactivation. Various hypotheses have been proposed to explain the formation of RhAlO_x_, including the disappearance of Rh nanoparticles or the aggregation into clusters following exposure to air aging.^[91]^ Li and his collaborators found direct evidence for the presence of RhAlO_x_ using scanning transmission electron microscopy (STEM), electron energy loss spectroscopy (EELS), and extended x‐ray absorption fine structure spectroscopy (EXAFS) (Figure 11d).^[87]^ They loaded 5% Rh on alumina by impregnation. They found that the high‐temperature deactivation mechanism of Rh/Al_2_O_3_ consists of two parts: the sintered metal Rh nanoparticles into Rh oxide particles and the formation of RhAlO_x_ by occupying octahedral sites on the (200) crystal plane of Al_2_O_3_. The reduction treatment of aged Rh/Al_2_O_3_ can restore part of the catalytic activity, and the renewability of the catalyst depends on the ability of RhAlO_x_ to be restored to Rh nanoparticles. Understanding the reaction mechanism and deactivation mechanism can deepen the understanding of Rh‐based three‐way catalysts. For the three‐way catalytic reaction of NO_x_ elimination, Rh is used for its unique NO_x_ adsorption ability, and the metal Rh often has higher catalytic activity. However, the metal‐support interaction often causes the metal Rh to be deactivated in harsh atmospheres. With our new understanding of the structure of Rh‐based three‐way catalysts, it is expected to achieve well‐designed, highly stable, wide temperature range and low‐cost Rh‐based three‐way catalysts in the future.

## 5 CONCLUSIONS AND OUTLOOK

Rh is widely used in three‐way catalysts due to its excellent NO_x_ elimination ability. Establishing a clear relationship between catalytic performance and Rh‐based catalyst fine structure is essential for advanced three‐way catalysts. This review describes the classification, catalytic mechanism, and deactivation mechanism of Rh‐based three‐way catalysts. In this review, we provide a comprehensive review of Rh‐based three‐way catalysts, and we summarize the important role of different supports as well as different active center structures for the catalytic activity, selectivity, and stability. To deepen the understanding of Rh‐based three‐way catalysts, we further introduced the mechanism of CO + NO reaction in detail and summarized several highly active Rh species and their inactivation mechanisms. Although Rh has an irreplaceable role in three‐way catalysts, it still faces many industrial problems that need to be solved urgently, such as high cost, insufficient low‐temperature activity, unavoidable sintering, and unclear reaction mechanisms. To meet this challenge, future research efforts are needed in the following areas:

(i) Constructing highly dispersed Rh active centers with low chemical valence. High dispersion is an effective way to increase the Rh utilization; however, the valence of Rh also increases with dispersion, resulting in a decrease in performance. It is a challenge to balance the chemical valence and dispersion. To address this problem, it may be feasible to fabricate stable ultra‐small Rh clusters or Rh alloys.
(ii) Improved reactivity of Rh‐based catalysts toward CO and HC oxidation. Although the Rh‐based catalysts have the advantage of NO reduction reaction, they are usually less active towards the other two substrates. The existing challenges are the insufficient adsorption capacity and sluggish kinetics. To comprehensively improve the reaction activity, the rational construction of multifunctional supports and active centers to avoid substrate competition adsorption may be a very promising strategy.
(iii) Define the reaction mechanism. Currently, the progress of mechanism research is slow due to the complexity of the actual environment of the three‐way reaction. Although the mechanistic study based on numerical simulations can explain the adsorption capacity from the perspective of active sites, in situ infrared and other spectroscopic experiments are still needed to identify the reactive intermediate species. Further understanding of the microscopic reaction mechanism is required to design Rh‐based three‐way catalysts with better performance.

We hope that the review in this paper will make an important contribution to the in‐depth development of Rh‐based three‐way catalysts or even highly active and low‐cost three‐way catalysts for the industrial application of automotive exhaust gas treatment.

## AUTHOR CONTRIBUTIONS

The manuscript was written through the contributions of all authors. All authors have given approval to the final version of the manuscript.

## CONFLICT OF INTEREST STATEMENT

The authors declare no conflicts of interest.

## Data Availability

The data that support the findings of this study are openly available.
